# S03-3 Staying active together in sports - the importance of and how to promote the emotional domain of Physical literacy

**DOI:** 10.1093/eurpub/ckac093.014

**Published:** 2022-08-29

**Authors:** Glen Nielsen, Peter Bentsen, Peter Elsborg

**Affiliations:** 1 Department of Nutrition, Exercise and Sports, University of Copenhagen; 2 Center for Clinical Research and Prevention, Copenhagen University Hospital – Bispebjerg and Frederiksberg; 3 Department of Geosciences and Natural Resource Management, University of Copenhagen, Copenhagen, Denmark

**Keywords:** Empowering coaching, motivation, physical literacy, sport participation

## Abstract

**Background:**

Club-based sports participation through adolescent years is associated with improved health and wellbeing and is therefore considered and important part of health promotion in Scandinavian welfare states. Participation rates are very high among children whereas dropout rates increase during the adolescent years. Many studies have shown how intentions to continue in sport is dependent on autonomous motivation, which is dependent on basic psychological needs satisfaction and hence needs support in the social environment of the team. It has also been shown that the coach has central influence on social environment of the team. The SATS study is a prospective study on the influence of coach-created social climate on young club-sport participants’ psychological needs satisfaction, motivation and continuation the following season.

**Methods:**

Participants were 6400 adolescent members of leisure time club-organized Basketball, Handball, Football and Gymnastics in Denmark. In the baseline-season coach-created climate was measured with the EDMCQ-C, Basic Psychological needs satisfaction and frustration was measured with PNSS-S and behavioral regulation (motivation) was measured with BRSQ. The participants’ continuation or dropout the next season was measured with a short SMS based questionnaire the next season.

**Results:**

Task oriented, social supportive and autonomy supportive coach behaviors were associated with higher basic needs satisfaction, autonomous motivation and continuation the next season across sports, ages, levels and genders. Ego-oriented and controlling coaching behaviors were associated with needs frustration, controlled motivation and dropout.

**Conclusions:**

Empowering Coaching behavior is important for young sports participants’ wellbeing and continuation in sport.

